# Impact of GLP-1 Receptor Agonists on Major Gastrointestinal Disorders for Type 2 Diabetes Mellitus: A Mixed Treatment Comparison Meta-Analysis

**DOI:** 10.1155/2012/230624

**Published:** 2012-12-26

**Authors:** Feng Sun, Kai Yu, Zhirong Yang, Shanshan Wu, Yuan Zhang, Luwen Shi, Linong Ji, Siyan Zhan

**Affiliations:** ^1^Department of Epidemiology and Biostatistics, School of Public Health, Peking University Health Science Centre, Beijing 100191, China; ^2^International Research Center of Medicinal Administration, Peking University, Beijing 100191, China; ^3^Department of Preventive Medicine, College of Medicine, Shihezi University, Shihezi 832002, China; ^4^Department of Orthopedics, Tianjin Fifth Central Hospital, Tianjin 300450, China; ^5^Department of Endocrinology and Metabolism, People's Hospital, Peking University, Beijing 100044, China

## Abstract

*Aim*. We aimed to integrate evidence from all randomized controlled trials (RCTs) and assess the impact of different doses of exenatide or liraglutide on major gastrointestinal adverse events (GIAEs) in type 2 diabetes (T2DM). *Methods*. RCTs evaluating different doses of exenatide and liraglutide against placebo or an active comparator with treatment duration ≥4 weeks were searched and reviewed. A total of 35, 32 and 28 RCTs met the selection criteria evaluated for nausea, vomiting, and diarrhea, respectively. Pairwise random-effects meta-analyses and mixed treatment comparisons (MTC) of all RCTs were performed. *Results*. All GLP-1 dose groups significantly increased the probability of nausea, vomiting and diarrhea relative to placebo and conventional treatment. MTC meta-analysis showed that there was 99.2% and 85.0% probability, respectively, that people with exenatide 10 **μ**g twice daily (EX10BID) was more vulnerable to nausea and vomiting than those with other treatments. There was a 78.90% probability that liraglutide 1.2 mg once daily (LIR1.2) has a higher risk of diarrhea than other groups. A dose-dependent relationship of exenatide and liraglutide on GIAEs was observed. *Conclusions*. Our MTC meta-analysis suggests that patients should be warned about these GIAEs in early stage of treatment by GLP-1s, especially by EX10BID and LIR1.2, to promote treatment compliance.

## 1. Introduction

Diabetes is a major public health problem. In 2000, there were 171 million patients with diabetes mellitus worldwide, and the number is predicted to increase to 366 million by 2030 [1]. As the number of people with diabetes has increased, so too has the availability of treatments for managing the disease. In recent years, glucagon-like peptide-1 agonists (GLP-1s) [2], as an innovative generation of antidiabetic drugs administered by injection under the skin, have been introduced into clinical practice and offer new possibilities for treating hyperglycemia in people with T2DM [3]. GLP-1s regulate glucose levels by stimulating glucose-dependent insulin secretion and biosynthesis and by suppressing glucagon secretion, delaying gastric emptying and promoting satiety [4–6]. Various GLP-1s are in use or in the licensing process, including exenatide [7], liraglutide [8], albiglutide [9], taspoglutide [10], lixisenatide [11], and LY2189265 [12]; these latter 4 drugs are now in Phase II or III clinical trials.

At present, the GLP-1s are routinely administered once or twice daily or once weekly. As for native GLP-1, the most frequently reported treatment-related adverse event (AE) about GLP-1s was gastrointestinal (GI) disorders, mainly including nausea, vomiting, and diarrhea; AE rate and severity are linked to treatment adherence, sometimes leading to discontinuation of the drug in some people. There is some evidence that the GI AEs associated with GLP-1s are dose-dependent and decline over time [13, 14]. However, it is unclear if the risk of GI AEs differs in people with diverse available doses and frequencies of these GLP-1s.

Therefore, in this context, we collected all RCTs of comparing two currently approved GLP-1s, exenatide and liraglutide, with placebo or traditional antidiabetic. Pairwise random effect meta-analyses were performed to compare the impact of different dosing of GLP-1s on GI AEs in T2DM patients, and an additional MTC meta-analysis, for the first time, was carried out to investigate the robustness of the pairwise meta-analysis, to combine both direct and indirect evidence, and to rank these treatments in terms of GI AEs.

## 2. Materials and Methods 

### 2.1. Search Strategy

In consultation with a medical librarian, we established a search strategy for the following three databases (from inception to Oct 31 2011): MEDLINE, EMBASE, and Cochrane library. The following search strategy (Ovid) was adapted for the other databases:

1. exp glucagon-like peptide-1 agonists/
2. (glucagon like peptide* or GLP-1).tw.
3. (exenatide or liraglutide).tw.
4. randomized controlled trial.pt.
5. (randomized or randomised).tw.
6. (1 or 2 or 3) and (4 or 5).

We also searched http://www.ClinicalTrials.gov/ for (unpublished) completed trials. In addition, we searched the bibliographies of published systematic reviews [15–18]. All relevant authors and principal manufacturers were contacted to supplement incomplete reports of the original papers or to provide new data for unpublished studies.

### 2.2. Data Extraction and Quality Evaluation

Four investigators in our review team (YK, WSS, ZY, and YZR) worked independently, in duplicate. They scanned all abstracts and then obtained the full text reports indicative of an RCT with adverse events reported. Studies had to compare a GLP-1 to placebo, standard therapy, or another GLP-1 in T2DM patients with duration of at least four weeks.

After obtaining full reports of the candidate trials (either in full peer-reviewed publication or press article), the same reviewers independently extracted information from full text papers using a standardized prepiloted form, including population characteristics (age, T2DM course, and baseline HbA1C) and GI AEs (including nausea, vomiting, and diarrhea). Quality of studies was assessed according to JADAD scale [19]: adequate method for randomization, appropriate blinding procedures, and detailed report of withdrawals. We resolved differences in extraction through discussion and consensus.

### 2.3. Clinical Dosage of GLP-1 Agent

We only included dosages that are likely to be used in routine clinical care. We excluded trials or arms using nonstandard doses, which mainly came from dose-ranging studies. So only those dose arms possibly relevant with clinical application were included in our study. The standard exenatide regimens are 5 *μ*g twice daily (EX5BID), 10 *μ*g twice daily (EX10BID) and 2 mg once weekly (EX2QW). The standard liraglutide regimens are 0.6 mg once daily (LIR0.6), 1.2 mg once daily (LIR1.2), and 1.8 mg once daily (LIR1.8), respectively.

### 2.4. Data Analysis

As every traditional pairwise comparison between GLP-1 drugs, for involving only dichotomous outcomes in our analysis, we calculated the odds ratio (OR) and appropriate 95% confidence intervals (CIs) for all relevant outcomes according to the number of events reported in the original studies or substudies intent-to-treat analysis. Where studies did not report intent-to-treat, we analyzed outcomes as all-patients randomized. In the event of zero outcome events in one arm of a trial, we applied the Haldane method and added 0.5 to each cell [20]. We pooled summary estimate using the DerSimonian-Laird random effects method [21], which recognizes and anchors studies as a sample of all potential studies. The *I* ^2^ statistic was calculated as a measure of the proportion of the overall variation that is attributable to between-study heterogeneity [22]. 

Second, in order to evaluate the relative effectiveness of each GLP-1 drug on GI AEs, we did a mixed treatment comparison (MTC) meta-analysis within a Bayesian framework [23, 24], and we summarized the results using OR and their CIs. Bayesian MTC meta-analysis is a generalization of traditional meta-analysis that allows all evidence to be taken into account simultaneously (both direct and indirect). It was proposed by Lu and Ades and can be applied whenever a connected network of evidence is available [23, 24]. The MTC results depend on the network of evidence and can provide narrower interval estimates. The models are based on the Bayesian hierarchical framework and are very flexible, allowing the incorporation of data characteristics like multiple-arm trials and heterogeneous between trials' variability. 

One key assumption of the MTC models is the consistency between direct and indirect evidence, that is, if the information of both sources of evidence is similar enough in order to be combined. To estimate inconsistency, we calculated the difference between indirect and direct estimates whenever indirect estimates could be constructed with a single common comparator [25]. Inconsistency was defined as disagreement between direct and indirect evidence with a 95% CI excluding 0. We estimated the posterior densities for all unknown parameters using MCMC (Markov chain Monte Carlo) for each model. Each chain used 40 000 iterations with a burn-in of 20 000. To formally check whether a model's overall fit is satisfactory, we consider an absolute measure of fit: ${\overset{-}{D}}_{\text{res}}$, the posterior mean of the residual deviance (the deviance for the fitted model minus the deviance for the saturated model). We would expect that each data point should contribute about 1 to the posterior mean deviance so that it can be compared to the number of data points for the purpose of checking model fit [26]. We calculated the probability for each GLP-1 drug to be the most harmful (first-worst) regimen, the second-worst, the third-worst, and so on, and presented the results graphically with rankograms and surface under the cumulative ranking curve (SUCRA), which is equal to 1 when the treatment is certain to be the best and 0 when it is certain to be the worst [27]. 

Analyses were conducted using *STATA 10.0* (pairwise meta-analysis and *I* ^2^ calculations), *R 2.13.1* (estimation of inconsistency, rankograms and SUCRA graphs), and *WinBUGS 1.4.3* (MTC meta-analysis, model fit).

## 3. Results

### 3.1. Study Characteristics and Methodological Quality

**Figure 1:**
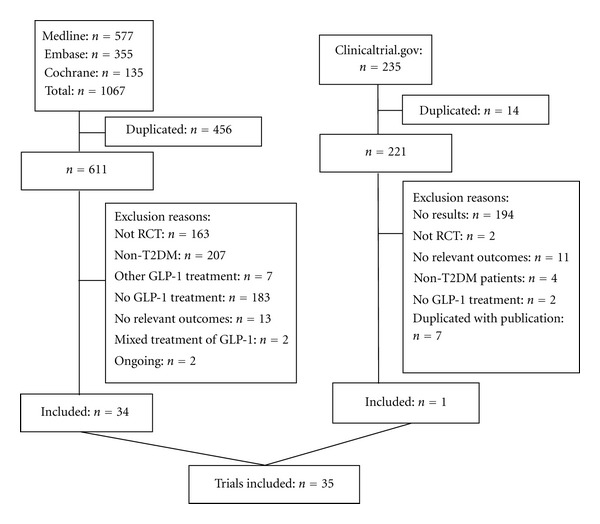
Flow diagram of included studies.

35 RCTs meeting inclusion criteria were identified for MTC meta-analysis (Figure 1). The range of publication year was 2002–2011.The average age of included participants was 55.63 years (standard deviation (SD) 2.09), ranging from 51.9 to 60.3. The range of duration of studies was from 4 to 104 weeks. The mean pre-treatment HbA1c level was 8.26% (SD 0.42%) and ranged from 7.3% to 9.3%. The average T2DM course was 7.10 years (SD 2.17), ranging from 2.0 to 12.0. Table 1 displays the study characteristics. 

We found that the reporting quality of studies varied. The overall quality of studies was rated as good according to JADAD scale; the proportions of appropriate description of randomization, allocation concealment, blinding and dropout were 74.29%, 57.14%, 60.00%, and 94.29% respectively. Additionally, 94.29% trials used intention-to-treat analysis. (see supplemental Table  1 in Supplementary Material available online at doi:10.1155/2012/230624).

### 3.2. Evidence Network

**Figure 2:**
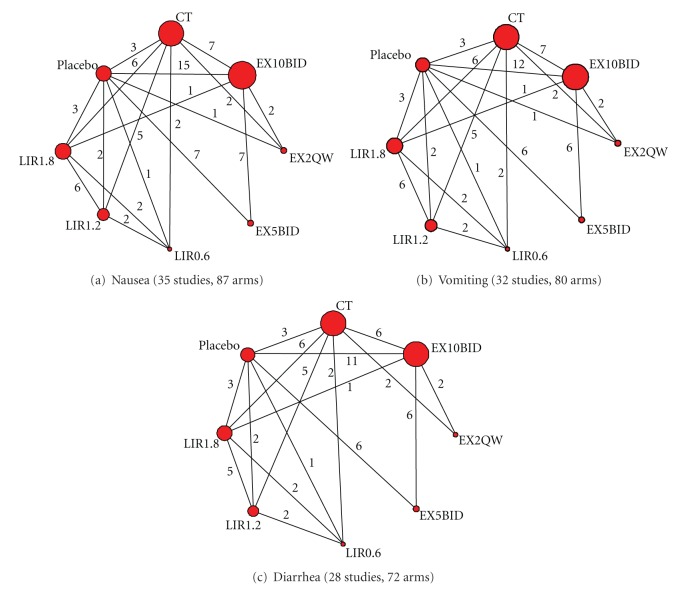
Evidence of structure of GI AEs for MTC meta-analysis. The numbers along the link lines indicate the number of trials or pairs of trial arms. Lines connect the interventions that have been studied in head-to-head (direct) comparisons in the eligible RCTs. The width of the lines represents the cumulative number of RCTs for each comparison, and the size of every node is proportional to the number of randomized participants (sample size). CT: conventional treatment. EX5BID: exenatide 5 *μ*g twice daily; EX10BID: exenatide 10 *μ*g twice daily; EX2QW: exenatide 2 mg once weekly; LIR0.6: liraglutide 0.6 mg once daily; LIR1.2: liraglutide 1.2 mg once daily; LIR1.8: liraglutide 1.8 mg once daily.

Eight treatments were analyzed: EX10BID, EX2QW, EX5BID, LIR0.6, LIR1.2, LIR1.8, placebo, and conventional treatment (CT and detailed drugs included can be learned from Table 1) of T2DM. Most trials (22 (62.86%) of 35) were two-arm studies and the rest 13 (37.14%) were multiple-arm studies (see Table 1). Figure 2 displays the geometric distribution of the RCTs evidence, and the largest number of RCTs was always conducted between EX10BID and placebo for three GI AEs. A total of 12810 patients contributed to the analysis of nausea (Figure 2(a), including 35 studies and 87 arms together). 32 studies of all included studies (Figure 2(b), including 12412 patients, 80 arms together) reported vomiting, while 28 (Figure 2(c), including 11632 patients and 72 arms together) reported diarrhea.

### 3.3. Impact of GLP-1 Dose on GI AEs by Direct Comparison and MTC Meta-Analysis

We assessed the impact of different GLP-1 doses on GI AEs by direct comparison and MTC meta-analysis.

#### 3.3.1. Nausea

Table 2 showed that all GLP-1 dose groups significantly increased the probability of nausea relative to placebo and conventional treatment, the range of significant ORs was from 2.89 (95% CI: 1.22~6.89, EX2QW versus placebo) to 6.10 (95% CI: 4.09~9.11, EX10BID versus placebo). Patients treated by EX10BID represented higher probability of nausea than any other GLP-1 dose group by MTC meta-analysis, the range of significant ORs was from 2.16 (95% CI: 1.00~4.67, EX10BID versus LIR1.2) to 3.19 (95% CI: 1.14~8.98, EX10BID versus LIR1.8). We found a highest incidence of nausea for those treated by EX10BID (37.13% (1174/3162), 95% CI: 35.44%  ~38.84%) versus placebo (9.36% (164/1753), 95% CI: 8.03%  ~10.82%) and with a significant association (OR = 6.10, 95% CI: 4.09~9.11, *P* < 0.001, *I* ^2^ = 59.50%). MTC meta-analysis found there to be a 99.2% probability to believe that EX10BID enables a higher proportion of patients to occur nausea than any other treatment group (see Table 3).

We also observed significant dose-response differences between GLP-1 dose groups. We can see that patients with EX10BID had 2.28 and 2.78 times higher risk of developing nausea than those treated by EX5BID and EX2QW. For three dose groups of liraglutide, patients with LIR0.6 presented a lower risk of nausea (OR = 0.61, 95% CI: 0.43~0.87) than those with LIR1.8. Accordingly, EX10BID had highest incidence of nausea (37.13%), followed by EX5BID (30.99%) and EX2QW (18.83%), while patients with LIR0.6, LIR1.2, and LIR1.8 had 12.47%, 19.76%, and 21.20% on nausea, respectively (see Tables 2 and 3).

In addition, subgroup analysis results from MTC meta-analysis by stratification of treatment duration revealed that EX10BID had a significant higher impact than placebo in any therapy course, and simultaneously we found that EX10BID had a descending risk before 26 weeks on nausea. For liraglutide, LIR1.2 also showed a descending risk with the treatment course, and there was a dose response association between three dose groups after 52 weeks from LIR0.6 (OR = 3.60, 95% CI: 1.22~12.13) to LIR1.8 (OR = 6.75, 95% CI: 2.47~22.15) (see Table 4).

#### 3.3.2. Vomiting

As displayed for nausea, all GLP-1 dose groups had significantly worse impact on vomiting than placebo and CT at the 0.05 level (see Table 2); the range of significant ORs was from 3.91 (95% CI: 1.35~11.32, EX2QW versus placebo) to 21.20 (95% CI: 1.27~354.20, LIR0.6 versus placebo). We found a highest incidence of vomiting for those treated by EX10BID (13.13% (388/2954), 95% CI: 11.94%  ~14.41%) versus placebo (2.01% (32/1594), 95% CI: 1.38%  ~2.82%) and with significant association (OR = 4.45, 95% CI 2.88~6.88, *P* < 0.001, *I* ^2^ **= 3.10%). MTC meta-analysis found there to be an 85.0% probability to believe that EX10BID enables a higher proportion of patients to occur vomiting than any other treatment group (see Table 3).

We observed two pairs of significant dose-response associations between GLP-1 dose groups, one was EX10BID versus EX2QW (OR = 1.92, 95% CI: 1.10~3.36, *P* = 0.022, *I* ^2^ **= 0%), and the other was LIR0.6 versus LIR1.8 (OR = 0.61, 95% CI: 0.38~0.97, *P* = 0.036, *I* ^2^ = 10.30%). Although patients treated by different doses of exenatide showed more negative impact when compared with different doses of liraglutide, no statistical significance was found. Accordingly, patients treated by EX10BID had highest incidence of vomiting (13.13%), followed by EX5BID (10.66%) and EX2QW (7.30%), while patients with LIR0.6, LIR1.2, and LIR1.8 had 7.61%, 8.22% and 8.92% of vomiting, respectively, (see Tables 2 and 3).

In addition, subgroup analysis by MTC meta-analysis revealed that both exenatide and liraglutide, generally speaking, had a descending risk of vomiting with the prolongation of treatment when compared with placebo. For three dose groups of liraglutide, a significant dose response association existed from LIR0.6 (OR = 30.14, 95% CI: 1.26~724.88) to LIR1.8 (OR = 63.82, 95% CI: 2.74~1513.23) when compared with placebo within the course of 12~26 weeks (see Table 4).

#### 3.3.3. Diarrhea

Like nausea and vomiting, all GLP-1 dose groups had significantly worse impact on diarrhea than placebo and CT at the 0.05 level (see Table 2); the range of significant ORs was from 1.83 (95% CI: 1.13~2.98, LIR0.6 versus CT, *P* = 0.015, *I* ^2^ = 15.8%) to 3.41 (95% CI: 1.29~9.00, LIR0.6 versus placebo, *P* = 0.013, *I* ^2^ = 0%). This analysis found no significant differences between different doses of GLP-1 in the incidence of diarrhea.

We found the first three higher incidences of diarrhea in those treated by LIR1.8 (12.52% (205/1638), 95% CI: 10.95%  ~14.22%), EX2QW (12.09% (81/670), 95% CI: 9.78%  ~14.86%) and LIR1.2 (11.94% (140/1173), 95% CI: 10.13%  ~13.93%), respectively. When comparing with placebo, from MTC meta-analysis, we observed the largest OR among different doses of GLP-1s wasLIR1.2 (OR = 2.92, 95% CI: 1.44~5.96). Simultaneously, Bayesian model found LIR1.2 with highest probability (78.90%) was considered with a higher risk of diarrhea than any other treatment group (see Table 3). 

In addition, subgroup analysis revealed that three dose groups (EX10BID, LIR0.6 and LIR1.8) represented a significant increase of risk of diarrhea when compared with placebo after 26 weeks of therapy, but there was a decreased tendency for those treated by LIR1.2 after 26 weeks of therapy (see Table 4).

### 3.4. Ranking of Different Dosing of GLP-1 on GI AEs

**Figure 3:**
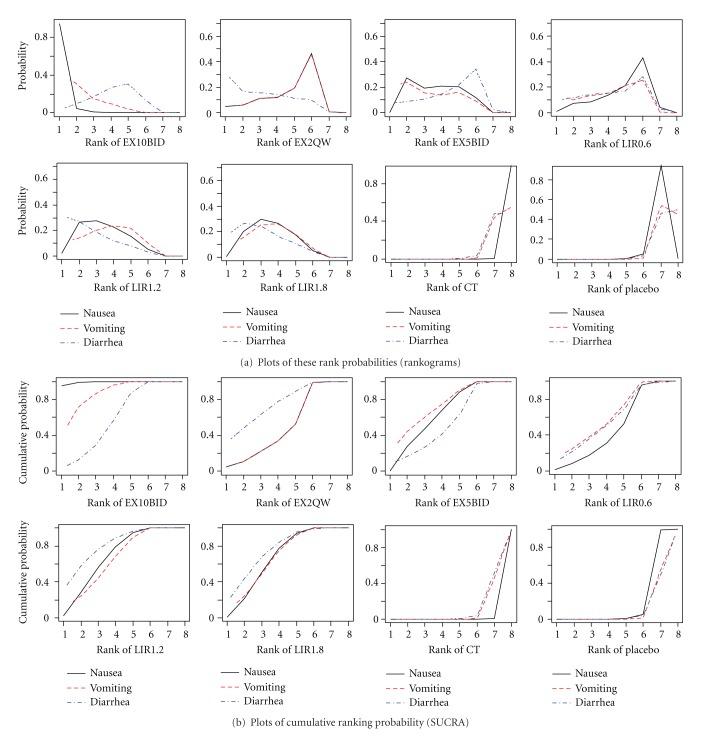
Plots for ranking probability of different dosing of GLP-1 on GI AEs. EX5BID: exenatide 5 *μ*g twice daily; EX10BID: exenatide 10 *μ*g twice daily; EX2QW: exenatide 2 mg once weekly; LIR0.6: liraglutide 0.6 mg once daily; LIR1.2: liraglutide 1.2 mg once daily; LIR1.8: liraglutide 1.8 mg once daily. Ranking: probability of being the worst treatment, of being the second worst, the third worst and so on, among the 8 comparisons. CT: conventional treatment. SUCRA: surface under the cumulative ranking curve. For rankogram, on the horizontal axis are the eight possible ranks and on the vertical axis the probability of a treatment to achieve each rank. For SUCRA plot, on the horizontal axis is the possible rank of each treatment (from the first best rank to worse according to the outcome). On the vertical axis is the cumulative probability for each treatment to be the best option, among the best two options, among the best three options, and so on.

Bayesian posterior probabilities can be used to rank the treatments for each outcome. Plots of these rank probabilities (see Figure 3(a), rankograms) are useful, but unlikely to provide an explicit ranking measure when many treatments are competing. A simple numerical summary to supplement the graphical display of cumulative ranking is to estimate the surface under the cumulative ranking (SUCRA, see Figure 3(b)) line for each treatment; SUCRA would be 1 when a treatment is certain to be the worst and 0 when a treatment is certain to be the best. SUCRAs plot and rankograms show the distribution of the probabilities of every treatment being ranked at each of the possible 8 positions.

Table 3 shows the mean SUCRA values for each outcome. According to SUCRAs, EX10BID had the most chance to have a negative impact both on nausea and vomiting, while for diarrhea, LIR1.2 had a 78.90% probability of having the highest impact of this outcome.

### 3.5. Model Fit and Inconsistence Check

The model fit can be evaluated using the posterior mean of the residual deviance ${\overset{-}{D}}_{\text{res}}$, we calculated the values of the ${\overset{-}{D}}_{\text{res}}$ for nausea, vomiting and diarrhea were 96.75, 90.21 and 83.60, respectively, which were close to corresponding 87, 80 and 72 of the number of data points for three GI disorders, meaning that model's overall fit is relatively satisfactory.

Additionally, statistical inconsistency between direct and indirect comparisons was generally low for three GI disorders. Most loops (networks of three or four comparisons that arise when collating studies involving different selections of competing treatments) were consistent, since their 95% CIs included 0 according to the forest plots, meaning that the direct estimate of the summary effect does not differentiate from the indirect estimate (see Supplemental Figure 1). Considering that the relatively low number of trials and events, relevant inconsistency from quadrilateral loops between trials could not be ruled out, many of the estimates from which were imprecise and do not allow for firm conclusions to be drawn from small sample size.

## 4. Discussion

Gastrointestinal complaints are commonly reported by diabetic patients. Previous studies indicate that about 70%–75% of diabetic patients have at least one gastrointestinal symptom [63, 64]. Some studies reported that inadequate glycemic control is the major cause of gastrointestinal symptoms [65–67]. Lack of glycemic control affects gastric motility, and delayed gastric emptying makes it difficult to control glucose levels, leading to gastrointestinal symptoms (early satiety, postprandial fullness, epigastric pain, nausea, and vomiting) in a vicious cycle process [65–67]. About the potential mechanism for the diarrhea, there are recent studies suggesting acceleration of colonic transit with another GLP-1 agonist, ROSE-010; in addition, the GLP-1 agonists may have effects on TGBAR receptor or other mechanisms that impact physiological secretion of bile acids. [68, 69]. Although these GI symptoms are not considered important causes of mortality in T2DM patients, they can also have a negative influence on diabetic control, diabetic complications, and health-related quality of life [70, 71]. Therefore, faced with the worldwide increase in the incidence and prevalence of T2DM [72], special attention should be given to the presence of gastrointestinal symptoms as an indication of T2DM complication in the population with T2DM.

GLP-1, an incretin hormone secreted in response to food intake, has been demonstrated to reduce appetite, food intake and body weight and to slow gastric emptying. The main adverse effects of GLP-1s are GI AEs, which appear to be dose related and could relate to its effects on gastric motor function, and antral distension in particular [73–75]. Exenatide and liraglutide, two approved GLP-1 receptor agonists in clinical practice, show the major dose-dependent adverse effects of nausea, vomiting, and diarrhea [76, 77].

In our MTC meta-analysis, all GLP-1 dose groups of exenatide and liraglutide significantly increased the probability of nausea, vomiting and diarrhea relative to placebo or conventional treatment. In the meantime, EX10BID among all GLP-1 dose groups always revealed the highest risk of both nausea and vomiting, when compared with placebo or conventional treatment. As well, patients with EX10BID had highest incidence of nausea (37.13%) and vomiting (13.13%) in contrast with other treatments, and patients with LIR1.8 had highest incidence of diarrhea (12.52%), indicating that exenatide and liraglutide had the more treatment-related GI AEs definitely than other treatment, especially EX10BID. In a latest review [77], author revealed that mild-to-moderate nausea was the most frequent adverse event with exenatide (36.9% versus 8.3% in the pooled comparator). An unrelated open-label extension study [78] of a 28-day trial reported that nausea and vomiting were the most common adverse effects with EX10BID for 26 weeks. In another 26-week, open-label, randomized, controlled trial [45] of 551 patients, GI AEs were reported more common in the exenatide group, including nausea (57.1%), vomiting (17.4%), and diarrhea (8.5%) than insulin glargine group. Norris et al. [15] reported in a systematic review that nausea was the most common adverse event in placebo- and active-controlled trials. In trials lasting 16–30 weeks, nausea was reported in 51% of subjects receiving exenatide + SU [34], 45% of subjects receiving exenatide + MET [37], 5% of subjects given exenatide + SU + MET [47], and 40% of those receiving exenatide + TZDs [54].

In terms of different dosing of GLP-1, by MTC meta-analysis, we found patients treated by EX10BID had 3.19, 2.16, and 2.24 times higher risk of developing nausea than those treated by LIR0.6, LIR1.2, and LIR1.8 respectively; for vomiting, patients treated by different doses of exenatide also showed more negative impact when compared with different doses of liraglutide, but no statistical significance. The results are similar to previous studies, which all showed that GI AEs were most pronounced with exenatide BID, 28% having nausea and 9.9% vomiting compared with 25.5 and 6.0%, respectively, during treatment with liraglutide [55, 79]. As nausea probably occurs at the peak of plasma concentrations of GLP-1 [80], the lower incidence of nausea with liraglutide compared with exenatide BID may be explained by its sustained release formulation and tachyphylaxia resulting from the sustained plasma level [55, 79, 81].

Additionally, the phenomenon of dose-related impact of exenatide and liraglutide on GI AEs was evident in our study. For three dose groups of exenatide, from MTC meta-analysis, we can see patients with EX10BID had 2.28 and 2.78 times higher risk of developing nausea than those treated by EX5BID and EX2QW. For three dose groups of liraglutide, patients with LIR0.6 presented a lower risk of nausea (OR = 0.61, 95% CI: 0.43~0.87) and vomiting (OR = 0.61, 95% CI: 0.38~0.97) than those with LIR1.8. Accordingly, EX10BID had highest incidence of nausea (37.13%) and vomiting (13.13%), followed by EX5BID (nausea: 30.99% and vomiting: 10.66%) and EX2QW(nausea: 18.83% and vomiting: 7.30%), while patients with LIR0.6, LIR1.2, and LIR1.8 had 12.47%, 19.76%, and 21.20% on nausea, 7.61%, 8.22% and 8.92% for vomiting, respectively, (see Table 3). The finding was also consistent with several previous studies [31, 34, 40, 46, 50, 58, 59]. Buse et al. [34], Moretto et al. [50] and Kadowaki et al. [46] observed a dose-dependent increase of nausea from EX5BID (range: 3~39%) to EX10BID (range: 13~51%); There is some evidence that exenatide used once weekly reduces this adverse event [31, 40]. Drucker et al. [40] and Blevins et al. [31] reported that EX10BID had more risk of nausea and vomiting than EX2QW. For liraglutide, Pratley et al. [59] and Nauk et al. [58] presented that there was a dose-dependent increase of GI AEs for three dose groups of liraglutide from LIR0.6, LIR1.2, to LIR1.8.

Our subgroup analysis by treatment course revealed that, with prolongation of treatment, patients treated by exenatide and liraglutide had a globally descending risk of nausea and vomiting when compared with placebo. But for diarrhea, no corresponding regular tendency was found. Several studies reported that these GI side effects occurred early on in the treatment, but tended to be transient and go away gradually after a few days or weeks [40, 55, 59, 62]. Buse et al. [55] reported that GI AEs were more common during the initial weeks of therapy. After 8–10 weeks the percentage of patients reporting nausea with liraglutide was below 10%, while in the exenatide group the level was over 10%; at the 26th week, only 2.5% of the liraglutide group had nausea compared with 8.6% in the exenatide group [55]. In LEAD-4 study [62], the incidence of nausea had decreased to the same level as in the placebo group after 16 weeks. This phenomenon told us that patients should be warned about these GI AEs especially in the initial stage of therapy, so that they are not taken by surprise to withdraw. If patients can tolerate these side effects, they will abate with time.

There are several strengths to consider in our analysis. First, our study is the largest evaluation of GLP-1s on GI AEs to date. Second, because the MTC meta-analysis complements traditional meta-analysis and systematic reviews, faced with multiple treatment options, allows dissection of the individual drug to evaluate GI AEs, especially faced with that very few RCTs have directly compared competing different dosing of GLP-1s in T2DM, we applied a Bayesian model to explore the effect of indirect comparison between them, which is thought to be the most appropriate method for multiple-treatments meta-analysis [23, 82]. Additionally, goodness of our model fit was relatively satisfactory, and we only found slight inconsistency among quadrilateral loops within evidence structure, so that the rank of all treatments based on posterior probability from Bayesian model can help decision makers to apply the rank of GLP-1 into practice. 

Several limitations need to be cautious. First, other unpublished literatures on relevant pharmaceutical websites were not searched and only trials in English were included, which may lead to a potential publication bias. Second, most trials included in this paper were not specially designed to evaluate GI AEs, with the risk of misdiagnosis and under diagnosis. Lastly, we did not investigate the distribution of clinical and methodological variables in detail that we suspected might be potential sources of either heterogeneity or inconsistency in every comparison-specific group of trials, although our pooled estimates were with the random effect approach and only had a slight inconsistency.

In summary, this MTC meta-analysis provides a useful and complete picture of the associations between GLP-1s, conventional antidiabetic drugs, and placebo on GI AEs. Overall, GLP-1s exert significantly more risk than placebo and conventional treatment on GI AEs. EX10BID and LIR1.2 compromise a higher proportion of T2DM patients with more probability in terms of nausea, vomiting, and diarrhea than any other treatment. We believe patients should be warned about these GI AEs when treated by GLP-1s, especially during the initial weeks of therapy by EX10BID and LIR1.2; these agents are not recommended in patients with severe gastrointestinal disease. These results should be considered in the development of clinical practice guidelines for improving the quality of life and prognosis in the medium and long term.

## Supplementary Material

Appendix figure 1: Forest plots of inconsistence check for all closed loops in MTC evidence (CT: Conventional treatment; EX5BID: exenatide 5 *µ*g twice daily; EX10BID: exenatide 10 *µ*g twice daily; EX2QW: exenatide 2 mg once weekly; LIR0.6: liraglutide 0.6 mg once daily; LIR1.2: liraglutide 1.2 mg once daily; LIR1.8: liraglutide 1.8 mg once daily)
Appendix table 1: Quality of included trials

## Figures and Tables

**Table 1:** Characteristics of the studies included in the MTC meta-analysis.

ID	Study (reference)	GI AE^§^	GLP-1 (*n*)	Control (*n*)	Background therapy	Trial duration (week)	Age (year)	T2DM course (year)	HbA1c0 (%)
1	Rosenstock et al. 2009 [28]	N, V, D	EX10BID (34)	Placebo (50)	Met	16	54	4.9	8
2	Apovian et al. 2010 [29]	N, D	EX10BID (96)	Placebo (98)	Met/Su/Su+Met	24	54.8	5.5	7.6
3	Barnett et al. 2007 [30]	N, V	EX10BID (138)	Insulin (138)	Met/Su	16	54.9	7.4	9
4	Blevins et al. 2011 [31]	N, V, D	EX10BID (123)	EX2QW (129)	Met+/−Su+/−TZD	24	55.5	7.0	NR
5	Bergenstal et al. 2010 [32]	N, V, D	EX10BID (160)	Pig (165) Sitagliptin (166)	Met	26	52.5	6	8.6
6	Bunck et al. 2009 [33]	N, V, D	EX10BID (36)	Insulin (33)	Met	52	58.3	4.9	7.5
7	Buse et al. 2004 [34]	N, V, D	EX5BID (125), EX10BID (129)	Placebo (123)	Su	30	55	6.3	8.6
8	Buse et al. 2011 [35]	N, V	EX10BID (137)	Placebo (122)	GLAR+/−CT	30	59	12	8.4
9	Davies et al. 2009 [36]	N, V, D	EX10BID (118)	Insulin (116)	Met/Su/TZD	26	56.5	8.7	8.6
10	DeFronzo et al. 2005 [37]	N, V, D	EX5BID (110), EX10BID (113)	Placebo (113)	Met	30	53	5.8	8.2
11	DeFronzo et al. 2010 [38]	N, V, D	EX10BID (45)	Rog (45)	Met	20	56	4.7	7.8
12	Diamant et al. 2010 [39]	N, V, D	EX2QW (233)	Insulin (233)	Met/Met+Su	26	58	7.9	8.3
13	Drucker et al. 2008 [40]	N, V, D	EX10BID (145)	EX2QW (148)	Met+/−Su+/−TZD	30	55	6.5	8.3
14	Fineman et al. 2003 [41]	N	EX10BID (81)	Placebo (28)	Met/Su	4	51.9	NR	9.3
15	Gallwitz et al. 2011 [42]	N, V, D	EX10BID (247)	Insulin (233)	Met/Su	26	NR	NR	NR
16	Gao et al. 2009 [43]	N, V, D	EX10BID (234)	Placebo (232)	Met/Met+Su	16	54.5	8	8.3
17	Gill et al. 2010 [44]	N, V, D	EX10BID (28)	Placebo (26)	Met/Met+TZD	12	55.6	6.5	7.3
18	Heine et al. 2005 [45]	N, V, D	EX10BID (282)	Insulin (267)	Met+Su	26	58.9	9.6	8.2
19	Kadowaki et al. 2009 [46]	N, V, D	EX5BID (37), EX10BID (37)	Placebo (40)	Su/Bg/Su+TZD/Bg	12	60.3	11.8	8
20	Kendall et al. 2005 [47]	N, V, D	EX5BID (245), EX10BID (241)	Placebo (247)	Met/Met+Su	30	55.3	8.9	8.5
21	Kim et al. 2007 [48]	N, V	EX2QW (15)	Placebo (14)	Met	15	54	5	8.5
22	Liutkus et al. 2010 [49]	N, V, D	EX10BID (111)	Placebo (54)	TZD/TZD+Met	26	54.7	6.4	8.2
23	Moretto et al. 2008 [50]	N, V, D	EX5BID (78), EX10BID (77)	Placebo (77)	None	24	54	2	7.8
24	Nauck et al. 2007 [51]	N, V, D	EX10BID (253)	Insulin (248)	Met/Su	52	58.5	9.9	8.6
25	NCT00577824, 2009 [52]	N, V, D	EX5BID (72), EX10BID (72)	Placebo (35)	None	24	58.4	NR	NR
26	Poon et al. 2005 [53]	N	EX5BID (31), EX10BID (31)	Placebo (33)	Met/none	4	52.9	3.9	7.6
27	Zinman et al. 2007 [54]	N, V, D	EX10BID (121)	Placebo (112)	TZD/TZD+Met	16	56	8	7.9
28	Buse et al. 2009 (LEAD6) [55]	N, V, D	LIR1.8 (235)	EX10BID (232)	Met/Su/Met+Su	26	56.7	8.2	8.3
29	Garber et al. 2009 (LEAD3) [56]	N, V, D	LIR1.2 (251), LIR1.8 (246)	Su (248)	None	104	53	5.4	8.3
30	Marre et al. 2009 (LEAD1) [57]	N, V, D	LIR1.2 (228), LIR1.8 (234)	Placebo (114) Rog (232)	Glimepride	26	56.1	6.5	8.4
31	Nauck et al. 2009 (LEAD2) [58]	N, V, D	LIR0.6 (242), LIR1.2 (240), LIR1.8 (242)	Placebo (121) Su (242)	Met	104	57	7.4	8.4
32	Pratley et al. 2011 [59]	N, V, D	LIR1.2 (221), LIR1.8 (218)	Sitagliptin (219)	Met	52	55.3	6.2	8.4
33	Russell-Jones et al. 2009 (LEAD5) [60]	V, D	LIR1.8 (230)	Placebo (114) Insulin (232)	Met & Glimepride	26	57.6	9.4	8.3
34	Yang et al. 2011 [61]	N, V	LIR0.6 (231), LIR1.2 (233), LIR1.8 (233)	Su (231)	Met	16	53.3	7.5	8.5
35	Zinman et al. 2009 (LEAD4) [62]	N, V	LIR1.2 (178)	LIR1.8 (178)	Met/Rog	26	55	9	8.5

^§^N: nausea; ^§^V: vomiting; ^§^D: diarrhea. EX5BID: exenatide 5 *μ*g twice daily; EX10BID: exenatide 10 *μ*g twice daily; EX2QW: exenatide 2 mg once weekly; LIR0.6: liraglutide 0.6 mg once daily; LIR1.2: liraglutide 1.2 mg once daily; LIR1.8: liraglutide 1.8 mg once daily. HbA1c0: baseline level of HbA1c. NR: not reported; Met: metformin; Bg: biguanide; Su: sulfonylureas; TZD: thiazolidinediones; Rog: rosiglitazone, Pig: pioglitazone; GLAR: insulin glargine; LEAD: liraglutide effect and action in diabetes.

**Table 2:** Summary of estimates of different GLP-1 dose on GI AEs by direct comparisons and MTC meta-analysis.

Comparators	Nausea OR (95% CI)		Vomiting OR (95%CI)		Diarrhea OR (95% CI)	
	Direct	MTC	Direct	MTC	Direct	MTC
EX10BID versus						
EX5BID	**1.90** **(1.36**, **2.65)**	**2.28** **(1.26**, **4.11)**	1.00 (0.61, 1.63)	1.20 (0.61, 2.38)	1.16 (0.71, 1.88)	1.07 (0.60, 1.89)
EX2QW	2.16 (0.98, 4.79)	**2.78** **(1.25**, **6.18)**	**1.92** **(1.10**, **3.36)**	1.88 (0.76, 4.67)	0.70 (0.31, 1.57)	0.82 (0.41, 1.64)
LIR0.6	**—**	**3.19** **(1.14**, **8.98)**	**—**	1.51 (0.49, 4.61)	**—**	1.02 (0.45, 2.32)
LIR1.2	**—**	**2.16** **(1.00**, **4.67)**	**—**	1.37 (0.58, 3.24)	**—**	0.77 (0.40, 1.49)
LIR1.8	1.14 (0.75, 1.71)	**2.24** **(1.11**, ** 4.51)**	1.74 (0.87, 3.47)	1.35 (0.63, 2.88)	0.98 (0.56, 1.70)	0.83 (0.47, 1.47)
CT	**19.36** **(10.41**, **35.98)**	**18.34** **(10.54**, **31.92)**	**5.52** **(3.70**, **8.23)**	**7.78** **(4.29**, ** 14.12)**	**2.23** **(1.54**, **3.23)**	**2.26** **(1.43**, **3.58)**
Placebo	**6.10** **(4.09**, **9.11)**	**8.05 ** **(5.15**, **12.57)**	**4.45** **(2.88**, **6.88)**	**7.35 ** **(4.03**, ** 13.40)**	**1.99** **(1.35**, **2.94)**	**2.25 ** **(1.45**, **3.51)**

EX2QW versus						
EX5BID	**—**	0.82 (0.31, 2.17)	**—**	0.64 (0.21, 1.97)	**—**	1.31 (0.54, 3.18)
LIR0.6	**—**	1.15 (0.34, 3.93)	**—**	0.80 (0.20, 3.16)	**—**	1.25 (0.46, 3.39)
LIR1.2	**—**	0.78 (0.28, 2.15)	**—**	0.73 (0.23, 2.33)	**—**	0.94 (0.40, 2.23)
LIR1.8		0.80 (0.30, 2.12)	**—**	0.72 (0.24, 2.14)	**—**	1.02 (0.45, 2.29)
CT	**5.07** **(1.43**, **18.04)**	**6.60 ** **(2.90**, **15.01)**	**4.26** **(1.83**, **9.90)**	**4.14 ** **(1.63**, ** 10.49)**	**2.23** **(1.33**, **3.74)**	**2.76 ** **(1.38**, **5.54)**
Placebo	2.18 (0.33, 14.36)	**2.89** **(1.22**, **6.89)**	0.94 (0.02, 50.31)	**3.91 ** **(1.35**, ** 11.32)**		**2.76 ** **(1.25**, **6.09)**

EX5BID versus						
LIR0.6	**—**	1.40 (0.44, 4.47)	**—**	1.26 (0.35, 4.56)	**—**	0.96 (0.36, 2.53)
LIR1.2	**—**	0.95 (0.37, 2.42)	**—**	1.14 (0.39, 3.33)	**—**	0.72 (0.31, 1.68)
LIR1.8	**—**	0.98 (0.41, 2.36)	**—**	1.12 (0.42, 3.01)	**—**	0.78 (0.36, 1.70)
CT	**—**	**8.05 ** **(3.69**, ** 17.56)**	**—**	**6.48 ** **(2.69**, **15.60)**	**—**	**2.12 ** **(1.04**, **4.31)**
Placebo	**3.41** **(2.08**, **5.57)**	**3.53 ** **(1.89**, ** 6.59)**	**3.76** **(2.25**, **6.28)**	**6.12 ** **(2.81**, **13.33)**	**1.90** **(1.22**, **2.96)**	**2.11 ** **(1.14**, **3.91)**

LIR0.6 versus						
LIR1.2	0.77 (0.53, 1.11)	0.68 (0.26, 1.77)	0.89 (0.56, 1.43)	0.91 (0.34, 2.39)	0.93 (0.61, 1.42)	0.75 (0.35, 1.60)
LIR1.8	**0.61** **(0.43**, **0.87)**	0.70 (0.27, 1.80)	**0.61** **(0.38**, **0.97)**	0.89 (0.34, 2.34)	0.70 (0.49, 1.01)	0.81 (0.39, 1.69)
CT	**4.28** **(2.23**, **8.22)**	**5.74 ** **(2.16**, ** 15.23)**	**7.53** **(1.85**, **30.72)**	**5.15 ** **(1.78**, **14.87)**	**1.83** **(1.13**, **2.98)**	**2.21 ** **(1.05**, ** 4.64)**
Placebo	**3.28** **(1.24**, **8.69)**	2.52 (0.88, 7.20)	**21.20** **(1.27**, **354.20)**	**4.87 ** **(1.45**, **16.35)**	**3.41** **(1.29**, **9.00)**	2.20 (0.94, 5.17)

LIR1.2 versus						
LIR1.8	0.86(0.51,1.45)	1.04 (0.56, 1.91)	0.79 (0.48, 1.30)	0.98 (0.52, 1.86)	0.76 (0.59, 0.99)	1.08 (0.63, 1.85)
CT	**4.91** **(2.68**, **8.99)**	**8.49 ** **(4.26**, ** 16.92)**	**4.43** **(2.01**, **9.76)**	**5.68 ** **(2.61**, **12.36)**	**1.93** **(1.34**, **2.78)**	**2.93 ** **(1.67**, ** 5.14)**
Placebo	**5.37** **(2.42**, **11.95)**	**3.73 ** **(1.67**, ** 8.30)**	**14.96** **(2.02**, **110.78)**	**5.36 ** **(2.01**, **14.33)**	4.80 (0.93, 24.77)	**2.92 ** **(1.44**, ** 5.96)**

LIR1.8 versus						
CT	**6.11** **(4.44**, **8.41)**	**8.20 ** **(4.39**, ** 15.31)**	**5.06** **(2.27**, **11.29)**	**5.76 ** **(2.93**, ** 11.36)**	**2.59** **(1.92**, **3.49)**	**2.71 ** **(1.67**, ** 4.41)**
Placebo	2.80 (0.65, 12.09)	**3.60 ** **(1.74**, ** 7.46)**	3.12 (0.48, 20.44)	**5.45 ** **(2.27**, ** 13.09)**	**2.82** **(1.35**, **5.89)**	**2.71 ** **(1.45**, ** 5.05)**

MTC: mixed comparison meta-analysis. GI: gastrointestinal. CT: conventional treatment. **—**: no available comparison. OR: odds ratio. OR > 1 means first treatment has more GI AEs. Significant associations are in bold. EX5BID: exenatide 5 *μ*g twice daily; EX10BID: exenatide 10 *μ*g twice daily; EX2QW: exenatide 2 mg once weekly; LIR0.6: liraglutide 0.6 mg once daily; LIR1.2: liraglutide 1.2 mg once daily; LIR1.8: liraglutide 1.8 mg once daily.

**Table 3:** GI AEs cumulative incidence and the probability that each treatment is associated with highest incidence.

Treatment	Nausea			Vomiting			Diarrhea		
	Incidence % (95% CI)	SUCRA	Rank	Incidence % (95% CI)	SUCRA	Rank	Incidence % (95% CI)	SUCRA	Rank
EX10BID	**37.13** **(35.44,** **38.84)**	**0.992**	1	**13.13** **(11.94,** **14.41)**	**0.850**	1	10.19 (9.09, 11.39)	0.555	4
EX2QW	18.83 (15.97, 21.96)	0.511	5	7.30 (5.47, 9.51)	0.463	6	12.09 (9.78, 14.86)	0.727	2
EX5BID	30.99 (27.57, 34.57)	0.616	4	10.66 (8.42, 13.26)	0.700	2	9.61 (7.48, 12.11)	0.504	6
LIR0.6	12.47 (9.63, 15.79)	0.437	6	7.61 (5.39, 10.38)	0.576	5	12.47 (9.63, 15.79)	0.544	5
LIR1.2	19.76 (17.67, 21.99)	0.658	2	8.22 (6.81, 9.81)	0.624	4	**11.94** **(10.13,** **13.93)**	**0.789**	1
LIR1.8	21.20 (19.34, 23.15)	0.635	3	8.92 (7.65, 10.33)	0.643	3	12.52 (10.95, 14.22)	0.726	3
CT	3.86 (3.19, 4.63)	0.001	8	2.19 (1.69, 2.80)	0.065	8	5.23 (4.42, 6.13)	0.079	7
placebo	9.36 (8.03, 10.82)	0.150	7	2.01 (1.38, 2.82)	0.080	7	4.97 (3.93, 6.18)	0.078	8

EX5BID: exenatide 5 *μ*g twice daily; EX10BID: exenatide 10 *μ*g twice daily; EX2QW: exenatide 2 mg once weekly; LIR0.6: liraglutide 0.6 mg once daily; LIR1.2: liraglutide 1.2 mg once daily; LIR1.8: liraglutide 1.8 mg once daily. Ranking: probability of being the worst treatment, of being the second worst, the third worst and so on, among the 8 comparisons. CT: conventional treatment. SUCRA: surface under the cumulative ranking curve.

**Table 4:** MTC meta-analysis results by stratification of treatment course showing the effect of different GLP-1 dose versus placebo on GI AEs.

GI disorder	Treatment	MTC estimate (95% CI) of different treatment course			
		≤12 weeks	>12 weeks	≥26 weeks	≥52 weeks
Nausea					

No. of studies		**4**	**12**	**14**	**5**
	Placebo (ref.)				
	EX10BID	**29.52** ** (2.89,** **301.80)**	**7.95** ** (3.30,** **19.17)**	**5.12** ** (2.16, ** **12.12)**	**123.84** ** (19.81,** **1145.96)**
	EX2QW	—	1.82 (0.42, 7.89)	3.53 (0.60, 20.61)	—
	EX5BID	6.87 (0.37, 128.05)	3.27 (0.72, 14.78)	**3.37** ** (1.12, ** **10.11)**	—
	LIR06	—	3.00 (0.22, 40.79)	—	**3.60** ** (1.22,** **12.13)**
	LIR12	—	3.31 (0.25, 44.56)	**10.08** ** (1.78,** **57.09)**	**5.45** ** (1.99,** **17.62)**
	LIR18	—	4.08 (0.31, 54.22)	3.68 (0.96, 14.14)	**6.75** ** (2.47,** **22.15)**
	CT	—	0.42 (0.09, 1.90)	0.30 (0.09, 0.99)	1.19 (0.42, 3.89)

Vomiting					

No. of studies		2	**11**	**14**	5
	Placebo (ref.)				
	EX10BID	1.97 (0.26, 15.15)	**17.25** ** (4.48,** **72.75)**	**4.07** ** (1.53,** **10.83)**	21.28 (0.72, 1095.54)
	EX2QW	—	**16.54** ** (1.69,** **167)**	1.87 (0.29, 12.12)	—
	EX5BID	—	5.92 (0.82, 42.91)	**4.51** ** (1.35,** **15.08)**	—
	LIR06	—	**30.14** ** (1.26,** **724.88)**	—	**17.90** ** (1.06,** **703.45)**
	LIR12	—	**39.92** ** (1.71,** **951.46)**	8.06 (0.73, 89.06)	14.43 (0.94, 506.23)
	LIR18	—	**63.82** ** (2.74,** **1513.23)**	3.81 (0.83, 17.61)	**15.77** ** (1.03,** **557.80)**
	CT	—	6.09 (0.79, 46.99)	0.44 (0.11, 1.69)	3.66 (0.2, 131.37)

Diarrhea					

No. of studies		1	10	**12**	**5**
	Placebo (ref.)				
	EX10BID	8.22 (0.39, 172.98)	1.95 (0.73, 5.19)	**2.13** ** (1.22,** **3.6)**	**8.76** ** (2.00,** **38.43)**
	EX2QW	—	4.09 (0.57, 29.47)	2.2 (0.85, 5.37)	—
	EX5BID	10.88 (0.54, 219.83)	1.99 (0.32, 12.30)	1.7 (0.94, 3.09)	—
	LIR06	—	2.49 (0.16, 39.90)	—	**3.73** ** (1.15,** **12.15)**
	LIR12	—	3.29 (0.20, 53.46)	**29.78** ** (6.91,** **150.05)**	**3.28** ** (1.11,** **9.75)**
	LIR18	—	3.73 (0.23, 59.56)	**2.53** ** (1.14, 5.28)**	**4.57** ** (1.53, 13.61)**
	CT	—	1.67 (0.20, 13.76)	0.85 (0.41, 1.66)	1.80 (0.61, 5.29)

EX5BID: exenatide 5 *μ*g twice daily; EX10BID: exenatide 10 *μ*g twice daily; EX2QW: exenatide 2 mg once weekly; LIR0.6: liraglutide 0.6 mg once daily; LIR1.2: liraglutide 1.2 mg once daily; LIR1.8: liraglutide 1.8 mg once daily. CT: conventional treatment. —: no available comparison; MTC: mixed treatment comparison.
