# Supplementary material for: Impact of GLP-1 Receptor Agonists on Major Gastrointestinal Disorders for Type 2 Diabetes Mellitus: A Mixed Treatment Comparison Meta-Analysis
Source: Exp Diabetes Res. 2012 Dec 26;2012:230624. doi: 10.1155/2012/230624 (PMC3540917; doi:10.1155/2012/230624)
Supplement: Supplementary file 1 — Appendix figure 1: Forest plots of inconsistence check for all closed loops in MTC evidence (CT: Conventional treatment; EX5BID: exenatide 5 µg twice daily; EX10BID: exenatide 10 µg twice daily; EX2QW: exenatide 2 mg once weekly; LIR0.6: liraglutide 0.6 mg once daily; LIR1.2: liraglutide 1.2 mg once daily; LIR1.8: liraglutide 1.8 mg once daily) Appendix table 1: Quality of included trials [file 230624.f1.docx]

**A. For nausea (Estimates with 95%CIs)**

**B. For vomiting (Estimates with 95%CIs)**

**C. For diarrhea (Estimates with 95%CIs)**

**Appendix figure 1. Forest plots of inconsistence check for all closed loops in MTC evidence**

(CT: Conventional treatment; EX5BID: exenatide 5μg twice daily; EX10BID: exenatide 10μg twice daily; EX2QW: exenatide 2mg once weekly; LIR0.6: liraglutide 0.6 mg once daily; LIR1.2: liraglutide 1.2 mg once daily; LIR1.8: liraglutide 1.8 mg once daily)
