# Supplementary material for: Impact of GLP-1 Receptor Agonists on Major Gastrointestinal Disorders for Type 2 Diabetes Mellitus: A Mixed Treatment Comparison Meta-Analysis
Source: Exp Diabetes Res. 2012 Dec 26;2012:230624. doi: 10.1155/2012/230624 (PMC3540917; doi:10.1155/2012/230624)
Supplement: Supplementary file 2 [file 230624.f2.docx]

**Appendix table 1 Quality of included trials**

| **ID** | **study** | **Clinical trial no** | **Adequate Sequence**  **generation** | **Allocation**  **concealment** | **Blinding** | **Dropout addressed** | **ITT** |
| --- | --- | --- | --- | --- | --- | --- | --- |
| 1 | Rosenstock J,2009[28] | NCT00518115 | Unclear | Unclear | Adequate,DB | Unclear | Yes |
| 2 | Apovian CM,2010[29] | NCT00375492 | Adequate | Adequate | Adequate,DB | Adequate | Yes |
| 3 | Barnett AH,2007[30] | NCT00099619 | Adequate | Adequate | Open- label | Adequate | Yes |
| 4 | Blevins T, 2011[31] | NR | Adequate | Adequate | Open- label | Adequate | Yes |
| 5 | Bergenstal RM,2010[32] | NCT00637273 | Adequate | Adequate | Adequate,DB | Adequate | Yes |
| 6 | Bunck MC,2009[33] | NCT00097500 | Adequate | Unclear | Unclear | Adequate | Yes |
| 7 | Buse JB,2004[34] | NCT00039026 | Adequate | Unclear | Adequate,DB | Adequate | Yes |
| 8 | Buse JB,2011[35] | NCT00765817 | Adequate | Adequate | Adequate,DB | Adequate | Yes |
| 9 | Davies MJ,2009[36] | NCT00360334 | Adequate | Unclear | Open- label | Adequate | Yes |
| 10 | DeFronzo RA,2005[37] | NCT00039013 | Unclear | Unclear | Adequate,TB | Adequate | Yes |
| 11 | DeFronzo RA,2010[38] | NCT00135330 | Adequate | Unclear | Open- label | Adequate | Yes |
| 12 | Diamant M,2010[39] | NCT00641056 | Adequate | Adequate | Open- label | Adequate | Yes |
| 13 | Drucker DJ,2008[40] | NCT00308139 | Adequate | Unclear | Open- label | Adequate | Yes |
| 14 | Fineman MS,2003[41] | NR | Adequate | Adequate | Adequate,TB | Adequate | Yes |
| 15 | Gallwitz B, 2011[42] | NCT00434954 | Adequate | Unclear | Open- label | NR | Yes |
| 16 | Gao Y,2009[43] | NCT00324363 | Adequate | Adequate | Adequate,DB | Adequate | Yes |
| 17 | Gill A,2010[44] | NCT00516074 | Unclear | Unclear | Adequate,DB | Adequate | Yes |
| 18 | Heine RJ,2005[45] | NCT00082381 | Adequate | Adequate | Open- label | Adequate | Yes |
| 19 | Kadowaki T,2009[46] | NCT00382239 | Unclear | Unclear | Adequate,DB | Adequate | Yes |
| 20 | Kendall DM,2005[47] | NCT00035984 | Unclear | Unclear | Adequate,DB | Adequate | Yes |
| 21 | Kim D,2007[48] | NCT00103935 | Adequate | Adequate | Adequate,DB | Adequate | Yes |
| 22 | Liutkus J,2010[49] | NCT00603239 | Adequate | Adequate | Unclear | Adequate | Yes |
| 23 | Moretto TJ,2008[50] | NCT00381342 | Adequate | Adequate | Adequate,DB | Adequate | Yes |
| 24 | Nauck MA,2007[51] | NCT00082407 | Adequate | Adequate | Unclear | Adequate | Yes |
| 25 | NCT00577824,2009[52] | NCT00577824 | Adequate | Unclear | Adequate,DB | Adequate | Yes |
| 26 | Poon T,2005[53] | NR | Unclear | Unclear | Unclear | Adequate | Yes |
| 27 | Zinman B,2007[54] | NCT00099320 | Adequate | Adequate | Adequate,DB | Adequate | Yes |
| 28 | Buse JB,2009 | NCT00518882 | Unclear | Adequate | Open- label | Adequate | Yes |
| 29 | (LEAD6)[55] | NCT00294723 | Adequate | Adequate | Adequate,DB | Adequate | Yes |
| 30 | Garber A,2009(LEAD3)[56] | NCT00318422 | Unclear | Unclear | Adequate,DB | Adequate | Yes |
| 31 | Marre M,2009(LEAD1)[57] | NCT00318461 | Adequate | Adequate | Adequate,DB | Adequate | Yes |
| 32 | Nauck M,2009(LEAD2)[58] | NCT00700817 | Adequate | Adequate | Open- label | Adequate | No |
| 33 | Pratley R,2011[59] | NCT00331851 | Adequate | Adequate | Adequate,DB | Adequate | Yes |
| 34 | Russell-Jones D, | NCT00614120 | Unclear | Unclear | Adequate,DB | Adequate | No |
| 35 | 2009(LEAD5)[60] | NCT00333151 | Adequate | Adequate | Adequate,DB | Adequate | Yes |

Note: ITT, intention-to-treat analysis. NR: not reported. LEAD: Liraglutide Effect and Action in Diabetes. DB: double blinding; TB: triple blinding.
